# The Hospital. Nursing Mirror

**Published:** 1898-09-24

**Authors:** 


### "The Hospital" Nursing Mirror.

Being the Nursing Section of "The Hospital."

[Contributions for this Section of "The Hospital" should be addressed to the Editor, The Hospital, 28 & 29, Southampton Street, Strand, London, W.C., and should have the word "Nursing" plainly written in left-hand top corner of the envelope.]

### News from the Nursing World.

### NEW ORDER FOR WOMEN.

In memory of the late Empress of Austria, the Emperor is about to establish a new Order for women. Medals will be struck bearing a representation of the Empress's patron saint, St. Elizabeth of Thuringia, and these will be awarded by the Emperor solely to those who behave in public and private life in such a manner as to preserve the dignity of the Order. The order will consist of three grades, with a Grand Cross for the first and second classes. The Cross of the first class will be of gold, enamelled in red; on one side will be the picture of St. Elizabeth, and on the other the letter E. The second class medal will be of the same design in silver. The first recipient of the Order is Countess Sztaray, who devotedly attended the Empress in her last hour.

### A LADY MILITARY SURGEON.

Miss Anita McGee is the first lady who has ever acted as military surgeon. She is commissioned as acting assistant surgeon in the United States Army. Her commission is for a limited period, to be renewed as her services are required. She is a member of the Red Cross Society. Miss McGee's first duty upon entering upon her post was to select 30 female nurses for the army in Porto Rico, who have been sent out as well as 12 male nurses. The nurses were chosen from the Icney Hospital, Brooklyn, and from the various training schools in Manhattan. The commission carries the rank, pay, and quarters of a second lieutenant, and the doctor will wear the same uniform, with a skirt of army cloth.

### THE ROYAL NATIONAL PENSION FUND FOR NURSES.

We beg to call attention to the fact that the amount of the total bonus distribution, in connection with the recent quinquennial valuation of this Fund, will be found in our advertising columns this week. Nurses who only desire to see the new prospectus to ascertain this fact may be glad of this information, as it will save them some trouble.

### SOUTHAMPTON NURSES.

The nurses of the Queen's Jubilee Nurses' Institute at Southampton will shortly go into their new home, the gift of Mr. A. Barlow. The advantages of living in one's own house are too obvious to need recapitulation, and they are augmented when the occupiers depend on public support. The new home is next door to the house already inhabited by the nurses in Bellevue Road, and the freehold cost the donor £553. The staff at present consists of the lady superintendent, two nurses, and a probationer; but in a town of some 100,000 inhabitants, so small a number of trained nurses must be inadequate, therefore the committee's appeal for more liberal support and more numerous subscriptions is fully justified.

### NURSES FOR THE PLAGUE.

It has become very evident to those who have had practical experience of nursing the Plague in India that those who undertake that work must not be too confident of receiving sympathy and support from those among whom their duties lie. This is instanced very strongly at the Macinktolla Plague Hospital, where it is stated that two Anglo-Indian nurses were dismissed to give place to two nurses from England. The Anglo-Indian Association have waxed very indignant, and have forwarded to the Bengal Government a resolution condemning the policy of the Government in letting native trained nurses be superseded by nurses imported from England.

### QUALIFIED RESIDENT DOCTOR.

The dividing line between doctors and nurses is, in theory, very sharply defined, and with excellent reason; nevertheless, in practice, circumstances often compel the nurse to act on her own responsibility, and the nursing profession is not without women who delight to do so, hence many of the annoyances and jealousies that arise between the disciples of the two branches of the healer's art. But nurses are not always to blame if they exceed their theoretical duties, for in institutions where there is no resident medical officer emergency cases happen more or less frequently, and the only choice left the nurse is to do what she can, or stand with folded hands until perhaps the time for acting has passed for ever. Complaint on this subject comes not unjustly from a parent at Newark. A mother accidentally gave her child a dose of liniment, and perceiving her mistake, at once took it to the hospital, only to find that no one could help her as there was no resident medical officer. The distracted woman was compelled to search the town for a doctor with her baby, in convulsions, in her arms. Such things ought not to be possible, for there are many ways in which the evil might be remedied. If a resident medical officer cannot be appointed, telephonic communication should be established with one or more of the visiting medical staff, with a proviso that one or other should always be within call; but to rule that nurses shall not usurp a doctor's duties, and then to set them in an institution for the reception of the sick without the power of invoking immediate medical aid in all emergencies, is illogical, to say the least of it.

### THE PRISONER'S RELEASE.

Mrs. Karl Neufeld, upon receiving information from the Government that her husband, who has been rescued after eleven years' captivity in Omdurman, was free, has resigned her post as matron of the Northwich (Cheshire) Rural Council's Hospital. She is making preparations to start for Egypt immediately, where she will await her husband, who returns with the British forces.

### CYCLING AND NURSING.

A CORRESPONDENT, in writing to us upon the topic of "Gentlewomen as Nurses," draws our attention to an advertisement for nurses who are non-cyclists. As the practice of bicycling for nurses is now not only recognised, but encouraged, at some of the largest and best administered hospitals in the kingdom, we agree with our correspondent that it would be interesting to know what originated the prohibition at the institution where the advertisement originated.

### BELFAST WORKHOUSE.

THE Guardians of Belfast Workhouse will not be persuaded to comply willingly with the order of the Local Government Board. Mr. Agnew, the inspector, reports that nothing has been done yet to improve the defective lavatory arrangements, and that the nursing staff is still inadequate. On the day of his visit one head nurse and one probationer and two paid pauper attendants were in charge of ninety-four inmates, of which number forty-four were lunatics or idiots. The pauper attendants have still to take their meals in the wards. The Local Government Board have sent a letter requiring the Guardians to appoint a trained night nurse, two paid attendants for the infirmary, and a paid attendant for the female infirm inmates. The entire surroundings of the place are insanitary, the cesspools are full, and are situated just under the windows of the typhoid wards. The Guardians convened a meeting, discussed the letter at length, and then declined to do anything. There is little to be gained by such obstinacy in wrong doing. Typhoid fever and lunatics are neither of them safe when they get the upper hand. The damage that can be done by a maniac or an imbecile is too horrible to contemplate in cold blood, whilst the ravages of typhoid fever are very costly. The action of the Local Government Board must have the approval of all sensible people.

### THE DEACONESS SOCIETY, OHIO.

THE eighth annual report of the Protestant Deaconess Society of Dayton, Ohio, lies before us. The term "deaconess" here implies a Protestant who spends her life in nursing the sick from a religious motive, and not for the sake of gain. This signification took its rise in Germany, when Pastor Fliedner adopted it in 1836 for the women enrolling themselves in the service of the sick under the influence of the movement inaugurated by his preaching. The result of this movement was the founding of the celebrated training school for nurses at Kaiserwerth, on the Rhine. The society at Dayton began work with two or three deaconesses in 1891, and a year later a temporary hospital was opened in Fourth Street. The nursing staff now consists of Miss Helen Heick and 26 deaconesses. Increased accommodation has been secured by making use of the basement for the dining-room, and the room used for this purpose being made into a sitting-room. No efforts are made to increase the number of nurses, but much care is taken to knit the members the one to the other, both in unity of purpose and thoroughness in work. Patients are received of all nationalities. Those who can pay are expected to do so, but all needy applicants for admission are attended free. It is somewhat significant that the number of pay patients during 1897 was 195, and that of free patients was 631.

### SISTERS AND GUARDIANS.

A CURIOUS hitch in the working of Poor Law institutions by religious sisterhoods has recently occurred in Middleton, near Dublin. The workhouse is nursed by the nuns of the Order of Mercy in Charleville, with Sister M. Bernard as matron. Some days ago Sister Bernard sent in her resignation, but the Guardians, expressing their sense of the value of her services, refused to accept it, referring the matter to the Sister Superior of the Order. She, however, has pointed out that the organisation of the Sisterhood compels her to make certain changes from time to time, and has nominated another Sister to the post. The Guardians have decided to request the Sister Superior to reconsider her decision; but she must of necessity place her duty to her community first, as all officers in a public appointment have the privilege of resigning if it pleased them to do so. The Guardians must be prepared to lose a satisfactory matron. This incident illustrates the difficulty of the dual interest which is perhaps the most difficult of solution in the problem of nursing by religious communities. The nun is first a religious devotee, and secondly a nurse; and hospital authorities are not unnaturally anxious to obtain the services of women who will be entirely under the control of the institution in which they work. There is much to be said on both sides.

### THE MOY MELL CHILDREN'S GUILD.

THE Moy Mell Children's Guild, which was organised some time ago in connection with the Children's Hospital, Temple Street, Dublin, was inaugurated one day last month at a meeting at the hospital. The Countess Cadogan was present, and was received by the Mother Superior, Sisters of Charity, and members of the medical staff, and an address in verse was recited by the son of Mr. M. M'D. Bodkin, Q.C. The object of the guild is to cultivate the sympathies of children with their suffering fellows; and it is hoped that this aim may be achieved by banding them together into "circles" under a secretary, and each circle contributing funds to the maintenance of a bed in the hospital.

### SHORT ITEMS.

THE Vicar of St. John's, Dorchester, and Mrs. Coote organised an entertainment in aid of the Parish Nursing Fund, which was very successful. The bicycle musical rides were much appreciated, and the manner in which the riders wove the Maypole ribbons and went through a set of lancers was exceedingly pretty. The money raised is said to have reached £11.—A wholesale increase of salaries has been granted to the officers of Newton Abbot Workhouse. The master's salary was raised to £80 a year, the matron's to £50, the porter's to £25, and the portress's to £15. A new male attendant was also engaged at a salary of £30, to assist the imbeciles and old men in feeding, bathing, and other toilet duties. The provision of uniform for the nurses and the granting to each of a three weeks' holiday in the year is to be considered at another time.—Miss Annie Varina Davis, daughter of Jefferson Davis, who led the Confederate States, died last Sunday at Rhode Island. She was well known as a writer under the names of "Winnie Davis" and "A Daughter of the Confederacy." She visited London a short time since.—By ten votes to nine the Thanet Board of Guardians have decreed that the nurses shall have a piano for their amusement. The nurses already have a tennis court, of which they have not greatly availed themselves.

### Nursing in Diseases of the Throat, Nose, and Ear.

By Macleod Yearsley, F.R.C.S.Eng., Assistant Surgeon to the Royal Ear Hospital, Surgeon-in-Charge of the Department for Diseases of the Throat, Nose, and Ear, the Farringdon General Dispensary, Hon. Surgeon for Diseases of the Throat and Ear, the Governesses' Home, &c.

### III.—GENERAL INSTRUCTIONS—(*continued*).

In passing on to those methods of treatment in the proper execution of which the nurse can be of great assistance, the necessity for thoroughness, combined with the utmost gentleness, cannot be too strongly impressed upon the reader. The ear, the throat, and the nose are organs of exquisite sensitiveness, and any roughness of manipulation not only disturbs the patient's confidence but defeats the object in view, and one cannot cultivate a more useful attainment than a gentle and delicate touch.

The most common duty the nurse is called upon to undertake is syringing. Simple proceeding as this is, it is curious how comparatively few individuals are adept at its practice. There is a "knack" about it not easily caught at first, but which once acquired is never lost. Instructions are not, therefore, out of place. The patient should be seated with the ear to be syringed opposite a good light. A towel is placed on the shoulder and tucked into the collar. A bowl, kidney-shaped for preference (the conical glass receptacle introduced by Gardiner Brown is very useful) is held under the ear against the neck by the patient himself, and if he does his part properly there is no need for the ear spouts and even more complicated arrangements which have been devised. The nurse then seizes the tip of the ear with the left thumb and forefinger, pulls it gently upwards and backwards to straighten the canal, and introduces the nozzle of the syringe into the opening. The point of the nozzle must not be directed straight inwards but towards the roof of the meatus, so that the fluid washes over the sensitive drum-head, and does not impinge directly upon it. No force need be applied whatever. After syringing the ear is dried with a soft towel, and the nurse should show the surgeon anything that has come away from the ear. It is not advisable to use plain water for syringing, water which has been sterilised by boiling, or boric acid lotion, carbolic (1 in 40), or corrosive sublimate (1 in 2,000) should be employed. Whatever fluid is used it must be made *comfortably warm.* The troubles for which the nurse will have to syringe are cerumen (ear-wax) or discharges. If she follows the instructions just given she should have no difficulty in removing the former, as the fluid, passing along the roof, washes out the plug of wax from behind. In syringing for pus a very gentle stream should be used.

In making local applications to the ear, instillations or "drops" are very frequently employed. They should be applied as follows: The patient lies upon his side, with the ear to be instilled uppermost. The drops, diluted if necessary, and properly warmed, are poured into the ear and retained (by the patient's position) for eight to ten minutes. A ready method is to use a teaspoon, in which the instillation can be easily warmed over the flame of a spirit lamp, and from which it can be conveniently poured into the ear. When the drops have been retained a sufficient time a pad of wool is placed over the ear to soak up the fluid as it runs out of the canal when the patient rises. On no account may drops be used cold.

Of other duties which the nurse may have to perform the application of blisters, heat, cold, or leeches, and packing the ear with antiseptic gauze are the most important.

Blisters are applied behind the ear over the mastoid process. The skin should first be cleansed with a little ether or ammonia to remove any fatty material, and then an area about the size of a shilling painted with liquor epispasticus; one layer is sufficient. When the blister rises it may be treated in the ordinary way.

Heat may be applied to the ear in a variety of ways. Poultices are not advisable, as they may cause severe inflammation (perichondritis) of the auricle. Dry heat by means of a large pad of hot cotton wool, or of hot flannel covered by wool and secured by a bandage, is the best method to employ. Heat may also be applied by instillations of sterilised water as hot as can be borne.

Cold is at times very useful. The ice bag is a good method of application, but Leiter's tubes are more convenient and effective.

Leeches form a very valuable means of treatment in diseases of the ear. Little need be said here as to the method of their application, a matter belonging to nursing in general, but it is not out of place to mention the spots at which they are usually applied in otology. These are: In front of the tragus, on the mastoid process, and occasionally beneath the ear.

Occasionally the nurse has to pack the ear with antiseptic gauze. This is done with double cyanide gauze, cut in strips from six to twelve inches long and half to one inch broad, soaked in 1 in 40 carbolic. One end is seized with a pair of aural forceps and gently passed into the meatus to the bottom; the remainder of the gauze is gently packed in, not too tightly, and the end curled up and lodged in the concha (or hollow of the ear). A pad of gauze, covered by a pad of wool and secured by a bandage, completes the dressing.

Passing now to the nose and throat, the nurse may have to assist in applying various external therapeutic measures to the latter similar to the applications used in other regions, and which need no special description, leeches, blisters, paints, compresses, poultices, &c. Cold is used by means of narrow ice-bags, cold compresses frequently changed, or by Leiter's tubes. Leiter's tubes are useful for applying both heat and cold; for the latter a temperature of 55 deg. F. suffices. For the application of fluids to the interior of the nose, sprays, atomisers, douches, or syringes are used. Douches are worked by a double hand-ball and are applied to the anterior nares or to the post-nasal space. The former is more frequently used, as, properly applied, it will reach the posterior nares efficiently. Leffert's directions for using the nasal spray are valuable and practical. I cannot do better than repeat them here. (1) Warm the fluid in the bottle by holding the latter for a few minutes in hot water; (2) let the body be held erect, the head very slightly inclined over a basin; (3) introduce the nozzle of the spray into the nostril, first into the one most occluded, far enough to close it perfectly, holding the tube of the apparatus directly outwards from the face without inclining it to one side or downwards. (4) Let the mouth be widely opened, breathing gently through it in a snoring manner and avoiding all attempts at speaking, swallowing, or coughing. The impulse to cough when the fluid passes into the upper part of the throat must be resisted, and it will pass into the opposite nostril. (5) The ball of the spray must be firmly grasped in the right hand and briskly worked until the fluid appears at the opposite nostril. (6) The nozzle must then be removed, and the superfluous fluid allowed to run out, the nose being blown gently, never vigorously. (7) The spraying should then be repeated upon the opposite nostril.

In spraying the pharynx the tube, preferably with a tongue depressor attached, should be introduced well into the mouth, and the spraying done by degrees to allow the patient to rid himself of the superfluous fluid.

Nasal douches are better used by the patient himself, but syringing may be done by the nurse. The syringe used may be an indiarubber handball syringe (best with a nozzle of the same material) or an ordinary Higginson's syringe. In using this method of cleansing the nose the following directions should be studied: (1) Warm the fluid. (2) Use no force. (3) Let the head be kept upright or inclined slightly forwards. If the head be thrown too much forward or back the fluid may enter the frontal air sinuses or the Eustachian tubes, with bad results. (4) The patient should breathe through the mouth, and avoid swallowing or coughing. (5) Syringe through the side which is most obstructed. (6) When first beginning do not use more than half a pint; at subsequent applications more may be employed. (7) Clear away superfluous fluid by gently blowing the nose.

Gargles, except by the method of Guinier, are not so frequently used as they were, and the nurse need not concern herself with them. Inhalations form a valuable method of locally treating diseases of the throat. They are best employed by means of Maw's Inhaler. The water used should be at a temperature of 140 deg. F. If an inhaler be not at hand, the treatment can be carried out with an ordinary jug. The jug should be about half full of water at the temperature named, and a folded towel arranged about its top in such a manner as to be adapted to the patient's nose and mouth. The inhalation should be continued for about ten minutes, the patient taking one breath of fresh air to every three or four of the vapour.

In painting the throat brushes should be discarded as uncleanly and inefficient. A pledget of cotton wool firmly held in a pair of bent catch-forceps should be dipped into the pigment and carried rapidly through the open mouth to the back of the throat, which should be quickly and thoroughly swabbed.

### Training in the Provinces.

(*Continued from page* 215.)

### VICTORIA INFIRMARY, GLASGOW (150 Beds).

### Terms of Training.

Probationers are received for three years' training at the Victoria Infirmary, Glasgow, between the ages of 24 and 32, and a certificate is granted on the satisfactory completion of the engagement and passing the required examination. Lectures are given to the nurses upon anatomy, physiology, &c., during their training by the assistant physician and surgeon. Probationers receive a salary of £12 during the first year, £16 the second, and £20 the third. Sisters' salaries begin at £30 per annum, increasing yearly by £2 to £40. Laundry expenses are defrayed by the hospital, and indoor uniform is also provided. A few paying probationers are received upon payment of a premium of £20.

Promotion to the higher posts on the staff are open to all probationers after three years' training, in accordance with their efficiency and suitability of character.

Night duty is taken by second year probationers for three months at a time in rotation.

### Hours On and Off Duty.

Nurses on day duty go their wards at 7.30 a.m., and leave them at 8.30 p.m. From these hours time for meals must be deducted, and off duty time, every second day from 2 to 4.30 p.m., other days, 4.45 to 6 p.m. In addition to this one evening weekly is given from 4.30 to 10 p.m., and one half-day every month, from 1 till 10 p.m. On Sundays time for church is allowed in the morning or evening, and "such other time as work will permit."

The sisters' hours on duty are between 8 a.m. and 10 p.m., with time allowed for meals, and off duty time every second day from 2 to 4.30 p.m., and on other days from 4.45 to 6 p.m. They have one whole day off duty once a month, and other times as the work of their wards will allow.

Night nurses are on duty from 9 p.m. to 9 a.m., with the usual daily morning off duty time, and one night off each month.

Annual leave is for probationers two weeks in the year, for staff nurses three weeks, and for sisters four weeks. The nurses are on duty in the morning of their "days off," but these days are sometimes given one or two at a time, so that an opportunity of going home is afforded.

### Meals.

All meals are served in the nurses' dining-room, with the exception of the 9.30 lunch and the tea at 2 p.m., which is taken either in the ward kitchen or in the sisters' room. No weekly allowances of food are made to the nurses themselves.

The hours for meals are as follows: Day nurses—breakfast at 7 a.m., lunch at 9.30 a.m., dinner 12.45 p.m., tea 4.15 to 4.45 p.m., and supper 8.30 p.m. Sisters' breakfast is half an hour later, they dine at 12.15 p.m., tea is served at 4.15, and supper at 8 p.m. The night nurses' breakfast is at 8.30 p.m., and their dinner at 9.30 a.m.

### GLOUCESTER GENERAL INFIRMARY (156 Beds).

### Terms of Training.

A month's trial is required of candidates who desire training at the Gloucester General Infirmary. They should be between 20 and 35 years of age, and must undertake to remain two years in the service of the hospital after completing eighteen months' training, the certificate being granted on completing the engagement of three and a half years. Probationers are not paid for the first eighteen months, salary commencing when on the expiration of the period of training they are placed on the nursing staff. As charge nurses, salary begins at £22 and rises to £32; as private nurses, at £20 rising to £30. Indoor uniform is provided by the hospital, and laundry expenses.

Weekly classes on nursing subjects are held. All probationers are alike eligible for promotion to the charge of wards, according to fitness and merit. Upon promotion to the staff nurses are required to join a pension fund. Eighteen months' training is obligatory before any nurse is so promoted, and it is usual to select the charge nurses from amongst the senior nurses on the private staff.

Night duty is taken for three months at a time by the probationers, under the supervision of a night charge nurse.

### Hours On and Off Duty.

Nurses are on duty between 7 a.m. and 9 p.m., with two hours daily for exercise and recreation, and time for meals. A whole day off duty is given every month to probationers when possible, and a whole day to charge nurses. A fortnight's holiday is allowed in the year.

### Meals.

The day nurses breakfast at 6.30 a.m., lunch is served from 10.15 to 11 a.m., dinner at 1.15 and at 2, tea at 4.30, and supper at 8.20 and 9. All meals are taken in the nurses' dining-room. Luncheon and tea are served for the nurses in the dining-room, the charge nurses taking these meals in their own rooms.

### A Book and its Story.

### RUSSIAN HOSTS AND ENGLISH GUESTS IN CENTRAL ASIA.

After reading this interesting account of Mr. Perowne's personally-conducted tour of a party of English people into Central Asia,* it is difficult to realise that until quite recently travellers who dared to venture in these regions carried their lives in their hands, or, at least, faced the gravest difficulties, while to-day the tourist is "not only permitted, but welcomed."

In the short space of eighteen years Russia has embraced in her iron grasp a territory that extends nearly 1,000 miles from east to west, and about 700 miles from north to south. "Like a crab she has stretched out her great claws, and slowly, but surely, she has closed them from east and west till they met in Merv. Wisely or unwisely," as Mr. Perowne points out, "we have allowed Russia to take this responsibility on herself," and throughout the volume he calls our attention to the vast amount of moral good that has resulted from her rule. "Had she done nothing more than sweep out of existence the horrors of a vile and universal slave traffic, civilisation would applaud. But under the skilful administration of the officers of the Czar, before long the Saturnian age of Central Asia must return. An empire worthy of Timur and Alexander is rising into existence."

Mr. Perowne's party for this memorable tour consisted of 25 travellers, who left the Pera Palace Hotel, Constantinople, last November, proceeding to Batoum by sea, which city they reached after a voyage of 87½ hours; considering that the distance thus traversed was not more than 700 miles, the speed of the boat was barely to be classified as express. On arrival at Batoum no difficulty was experienced by the party at the Custom House. By command of the Governor-General at Koutais none of the luggage was examined, an auspicious commencement of the goodwill shown throughout the tour by the Russians to the English travellers in Central Asia. From Batoum they proceeded to Tiflis, which city, to quote Mr. Perowne's words, "though about 1,500 feet above the sea level, is not romantically situated. A high, bare ridge shuts it in from the west. From this ridge the ground runs east to the watercourse of the Koura, and again rises on the farther side in an ugly brown bare slope, dotted with barracks and military store buildings, while south lies an open, uninteresting plain; north, far away, rise the snow mountains of the Caucasus. . . . The interesting parts of the city lie to the south, where are the Georgian, Persian, and Armenian quarters."

The account of the great petroleum country round Baku, which was next visited, is full of interest. Here in this vicinity are lakes of salt and of crude petroleum, and reservoirs full of the precious liquid. "Oil is, of course, the motive power of everything out here," writes Mr. Perowne, ". . . engines on the railway, steamers on the Caspian seas, machinery in the towns." This, indeed, is the veritable land of petroleum, a perfect sea of which underlies the country northward to the Caucasus, the very streets, in the dry weather, being watered by it. "Until a few years ago there was a remnant left of the old Zoroaster fire worship at Surakhanè, but the last priest was little better than a fraud, who lived on the charity of the curious, and now the temple is deserted, and the cult, founded over 2,500 years ago, which drew thousands of pilgrims annually from India, flickered out in the last half of the nineteenth century."

Throughout their tour Mr. Perowne's party met with the most marked attention and courtesy from the highest Russian authorities wherever the travellers were located. On arrival at Ashabad, a state visit was paid by the wanderers to the residence of General Kouropatkine, who had especially befriended the party. The General, who is one of Russia's most distinguished sons, and "more powerful than any English official in India, not even excepting the Viceroy, is paid the magnificent sum of £400 a year! But the Government of Central Asia makes an annual call on the Imperial Treasury of an enormous sum every year. These newly-acquired provinces of the empire are very far indeed from paying their way," and, as our authority urges, "this fact alone, of the heavy drain on Imperial sources from these Central Asia provinces, may calm the fears of English alarmists who think Russia is on the march to India."

The belief that the advance of Russia in Central Asia is merely a means to the final conquest of India is only too prevalent. Mr. Perowne's tour has led him to believe that her advance is merely an example of the extension of frontier that must necessarily take place when a civilised power "marches" with uncivilised tribes. This compulsory advance we have ourselves experienced in India, and it is consoling to know that Russia carries with her in her progress civilisation, law, and order. Probably when such tours as Mr. Perowne has just led become more common, more of us will share his belief that there is room enough in Central Asia for both England and Russia without interference with each other's objects.

But to return to the visit to General Kouropatkine, Mr. Perowne's party had every honour and hospitality shown them. "No political mission of the highest rank could have been treated better than we were." At Merv a wonderful exhibition of horsemanship was witnessed—a "Jigitofka," as it is called—which was held in honour of the English travellers. "One often reads of the wonderful horsemanship of the Cossack soldiers. . . . There was absolutely nothing," says the writer, "that they did not seem capable of doing with their horses going at full speed."

Of Merv—the old Merv—the Merv of history, called by poets and historians "the queen of the world," Mr. Perowne gives us a vivid account, but he adds: "The traveller who expects to see ruins such as those of Greece or Rome will be bitterly disappointed as he drives across the waste." "Our drive," he continues, "lasted about four hours, during which we drove through a desolating wilderness of ancient crumbling walls and gateways. . . . A desolation of miles of shapeless mounds and enormous brick city walls do not raise much emotion in a Westerner's heart. . . . The remains of three cities lie here side by side. . . . One could not help thinking what a magnificent field was here for antiquarian exploration and research. Bokhara and Samarcand are each described at some length as the merits of their individual interest entails. Of the former Mr. Perowne says: "To those who have only visited the Europeanised East, Bokhara is a revelation. . . . Here, too, is the residential palace of the Emir, though the Emir is reported to have but little liking for his capital." Of both these cities some excellent pictures illustrate the chapters dealing with them, illustrations which for their beauty of reproduction and interest form a fitting accompaniment to the pages of Mr. Perowne's delightful book, which, as he modestly puts it, he offers to his Russian hosts and friends as a token of his sense of gratitude for all that was done by them on behalf of the English travellers, "in order that some monument, however humble, might exist recording in how hospitable a manner a party of English people has been received by our traditionary foe in a far-off part of the world where that foe is supposed to be more inveterate and more insidious and more dangerous than anywhere else!"

* "Russian Hosts and English Guests in Central Asia." J. T. Woolrych Perowne. (London: The Scientific Press. 1898.)

### The Nurse's Reply to "A Sister's Lament."

WE like to please the Sisters,
But we nurses often find
That though they may not mean it,
They are really most unkind.

They expect from their probationers
What we're sure they'll never find,
That is, what they've ne'er taught them,
But they hope they'll bear in mind!

Forgetting they were students
Not so very long ago,
And made mistakes and blunders
Just like any other pro.

Their charts were just as wondrous,
And made their Sisters stare,
For everyone must live and learn,
Both in nursing and elsewhere.

The Sisters have so many pro.'s
To train from year to year,
They'll tell one how to do a thing,
Then forget which one, we fear.

Then when a nurse, through ignorance,
Does a thing that's not quite right,
The Sister tells her sternly,
"I showed you how last night!"

The poor girl racks her weary brain,
But cannot own 'tis so.
How can she, when she wasn't told?
It was another pro.

And so we ask the Sisters
To be patient with their pro.'s,
And remember which wants telling
And which already knows.

### Minor Appointments.

CROSSLAND MOOR WORKHOUSE INFIRMARY, HUDDERSFIELD. —Miss AdaRockett has been appointed Superintendent Nurse at the above infirmary. She was trained at the Leeds General Infirmary, Queen Charlotte's Hospital, and acted as assistant matron at the British Lying-In Hospital, Endell Street, and as home sister at the Central Nursing Home, Hull.

BRAINTREE COTTAGE HOSPITAL.—Miss Alice Rayner has been appointed Nurse-Matron of this cottage hospital. She was trained at St. Luke's Hospital, Halifax, and was afterwards charge nurse at the Manchester Consumption Hospital, the Stockwell Fever Hospital, and the Bradford City Hospital.

MANCHESTER ROYAL EYE HOSPITAL.—Miss Adah Stronghill has been appointed Ward Sister at this hospital. She was trained at University College Hospital, where she acted as staff nurse. She worked at the Hampstead Infirmary, and was matron of the Fleet Cottage Hospital.

UNION HOSPITAL, NEWCASTLE ON-TYNE.—Miss Louisa Clarke has been appointed Charge Nurse to this institution. Miss Clarke was trained at Town's Hospital, Glasgow, where she has been for three years, and is now proceeding to Newcastle.

SMALLWOOD HOSPITAL, REDDITCH.—Miss Ada Waller has been elected Matron of this hospital. She was trained at the Bradford Children's Hospital. She afterwards acted as charge nurse there, and at the Smallwood Hospital.

### Appointments.

MATRONS.

BOLTON PRIVATE NURSING HOME.—Miss Marion Clutterbuck has been appointed Lady Superintendent to this home. Miss Clutterbuck trained at the Royal Berks Hospital, Reading, where she was afterwards sister. She has also held appointments as sister at the Stafford General Infirmary, Marylebone Infirmary, London, and of assistant matron at the Salop Infirmary, Shrewsbury. For the past 2½ years Miss Clutterbuck has held the post of home sister to the Belfast Nurses' Home and Training School.

NEW FEVER HOSPITAL, HAM GREEN, BRISTOL.—Miss Mary Gardner has been appointed Matron of the above institution. She was trained at King's College Hospital, was for two years night sister at Princess Alice Hospital, Eastbourne, three years sister at the Staffordshire County Infirmary, night superintendent at the North-Western Fever Hospital, Hampstead, and was engaged in private nursing for a few months in Aberdeen.

FOREST INFECTIOUS HOSPITAL, MANSFIELD.—The vacancy of Matron at this institution has now been filled by the appointment of Miss L. S. Cooper, the head nurse at Barking Upney Isolation Hospital. Miss Cooper was trained at the Borough Hospital, Burton-on-Trent, and was for four years at the Isolation Hospital, Nottingham.

NURSING ASSOCIATION AND CONVALESCENT HOME, WOTTON-UNDER-EDGE.—Miss M. J. Tayler has been chosen to fill the post of Matron to this home. Miss Tayler has spent all her nursing life at the Bristol General Infirmary, where she was trained and where she has been for eleven years.

FORFAR INFIRMARY.—Miss Smith, the present nurse, has been appointed to the position of Matron, rendered vacant by the resignation of Miss Adams. Miss Smith has been nurse at the Forfar Infirmary for five years.

INFECTIOUS DISEASES HOSPITAL, KING'S NORTON.—Miss Mary Cooper has been appointed Matron to this institution. Miss Cooper has until recently been charge nurse and midwifery nurse at Selly Oak Workhouse Infirmary.

### Death in Our Ranks.

WE record with regret the death of Nurse Mary Ann Wetherall, who worked on the nursing staff of the Norfolk and Norwich Hospital Trained Nurses' Home. She died of blood poisoning contracted in the discharge of her duties. Nurse Wetherall received her training at St. Mary's Hospital. Her gentleness and consistent Christian life were much appreciated by her patients and those with whom she worked. Her remains were removed to her home in York and interred on September 13th.

### Presentations.

MISS BELLAMY, matron of the Infectious Hospital, Stockton-on-Tees, was presented by the nursing staff with a case of silver spoons and sugartongs, and with two knives from the domestic staff on resigning her post as matron. She carries with her the good wishes of her staff.

### Wants and Workers.

[THE attention of correspondents is directed to the fact that "Helps in Sickness and to Health" (Scientific Press, 28 & 29, Southampton Street, Strand, London, W.C.) will enable them promptly to find the most suitable accommodation for difficult or special cases.]

WILL any fellow-nurse offer a country home for the winter to a widowed nurse's daughter, aged 15, who is threatened with consumption? She has been working for Civil Service examination, and her health has broken down. Particulars from Mrs. E., 62, Salcott Road, Wandsworth Common, S.W.

### Everybody's Opinion.

[Correspondence on all subjects is invited, but we cannot in any way be responsible for the opinions expressed by our correspondents. No communication can be entertained if the name and address of the correspondent is not given, as a guarantee of good faith but not necessarily for publication, or unless one side of the paper only is written on.]

### LADIES *V.* GENTLEWOMEN.

"JUSTICE" writes: This subject has been well threshed out by your numerous correspondents, and those who, like myself, have taken no part in the controversy, must feel that both parties really agree in principle in spite of their apparent contradictions in application. Want of clear definitions always leads to much confusion, which a few words may make plain. The position conferred by birth, and the advantages derived from education and leisure, have given those so happy as to possess them, a social status not enjoyed by all women. The terms ladies or gentlewomen are used to denote such. There is, however, another nobility bestowed by nature, which is the heritage of all those who truly seek it. The portals that lead to it are lowly, and she who enters them must leave behind selfishness and all that appertains thereto, and be content to be nameless, for there is no title by which she can be known. It may be that, in spite of the many examples to the contrary, ladies in station have used the advantages of leisure and affluence in cultivating qualities that are regarded as essential to their position; but, however that may be, regarded from a Christian standpoint, the higher the station the greater the responsibilities. The possessor of wealth is not justified in being either a miser or a spendthrift. Neither has anyone the right to set at naught, or to use for her own gratification, the gift of position of which she is the steward.

"LIVERPOOL" writes: Suppose we define the words—gentlewoman, lady, good breeding, and education—as the dictionary gives the definitions? Gentlewoman: A woman of good family or good breeding and education, a term of civility to a female. Lady: A woman of distinction, a title of respect; any woman of refined manners and education. Good breeding: Polite manners, formed by good education, a polite education. Education: The bringing up of a child, instruction, the training that goes to cultivate the powers and form the character. These four words with their definitions explain the discussions recently published in THE HOSPITAL. Any woman can be a gentlewoman, and there are many such of to-day who are, as "Blue Blood" describes them, practically of no education save that of a Board school. "Blue Blood" has evidently not had any insight or experience of the "Board School education." For a thorough grounding in the three R's, geography, grammar, drawing, drill and music (tonic-solfa and theory), the advanced classes in physiology, elementary Latin, Greek, French, and German, and other subjects too numerous to mention, go to the Board schools. What is society? Would we call the society that "Blue Blood" apparently moves in always gentle? When we speak here of gentlewomen we do not mean those high in the social scale, but women who possess patience, good temper, kindness, generosity, humility, courteousness, unselfishness, guilelessness, and sincerity. Drummond says these virtues make up the supreme gift, the stature of the perfect man. Carlyle said of Robert Burns that there was no truer gentleman in Europe than the ploughman poet. He had not birth, but he had education and manners. "Blue Blood," in criticising "Brighouse's" experience of ladies, says, "Surely 'Brighouse's' experience with ladies must be very limited, or she must have come in contact with very questionable ladies." Is this the criticism of a lady or a gentlewoman? I venture to say, "No." Where is the charity that is kind? A gentlewoman has fine feelings, and studies the feelings of others, and this, to my mind, "Blue Blood" has failed to do here. A good and noble woman is a gentlewoman, and, if she is not born within the "pale of the society" in which "Blue Blood" moves, she inherits from Nature that of which she cannot be deprived. A well-bred racehorse sometimes does his breeding no credit. So fails also a well-bred cart-horse. Both require care, training, and attention besides the birth, and it is the same with individuals. Birth alone does not necessarily create a lady or a gentlewoman. Education in learning and manners, natural instincts, and home training all help to make the true gentlewoman. We find many of these women high in the social scale, with birth and education, and amongst the middle classes, with education, home-training, and natural instincts. Swift writes: One principal point of good breeding is to suit our behaviour to the three degrees of men—our superiors, our equals, and those below us. Chesterfield remarks: Good breeding is the result of much good sense, some good nature, and a little self-denial for the sake of others, and with a view to obtain the same indulgences from them. Sir P. Sydney says: A noble heart, like the sun, showeth its greatest countenance in its lowest estate. Sir Walter Scott quotes neither birth nor education for the nurse in these lines:—

> "Oh, Woman! in our hours of ease,
> Uncertain, coy, and hard to please;
> When pain and anguish wring the brow,
> A ministering angel thou."

And are we not rightly named ministering angels where the feelings of other nurses come in—poor angels!

"A MATRON" writes: If "A Doctor's Son" had given his name and address I would have written to him personally to thank him for his letter, which is in refres ing contrast to the snobbery and arrogance of some previous writers. I beg to assure him that, so far as my own experience goes, his sentiments *re* nurses and "gentlewomen" or "ladies" are shared by all who are, in the fullest sense of the word, the "best" nurses. I protest, not so much against the limitation of the use of those much-abused terms, for as matters stand they express certain conditions generally understood; but I do protest with deepest indignation against the concluding sentence of the letter of "A Doctor's Daughter": "I, for one . . . shall hail the day when the last 'servant class' nurse (however gentle and refined) is refused admission to our splendid hospital wards. This must be done if the reputation of the British nurse is to be maintained"! Can intolerance go further? I rejoice to think that the day anticipated will never come, since the tendency of the present age is rather in the direction of greater liberty and the breaking down of barriers of caste. That nursing is one of the most democratic professions has always been to me a source of peculiar pride and pleasure. Moreover, the combination of qualities necessary to make a really good nurse is not so common that we can afford to narrow the field by excluding any class. If "A Doctor's Daughter" is ever seriously ill she may have cause to appreciate the immeasurable difference between those who are nurses indeed and others who have merely learnt the technical part of their profession. It is difficult to summarise in brief the necessary qualifications of a nurse, because the work makes such all-round demands, but pre-eminent is the gift of sympathy, *i.e.*, practical sympathy, the power to put oneself in the place of another, to realise his condition and anticipate his needs, which is the outcome of an unselfish nature. I do not undervalue gentle birth and culture, but these are simply not essentials. If we can have them, together with the essential qualifications, so much the better. The nurse who is well born and educated has advantage over another less favoured; nevertheless, given the true raw material, nursing itself will prove an education. It has a refining, softening effect, very noticeable in those who undertake it in the right spirit, but it is no fanciful idea that the spirit is all-essential. For this work by its very nature, by the familiarity with suffering, and the sad initiation into life's darkest secrets which it brings, must either ennoble or debase, refine or coarsen, according to the mind's original bent. Our calling is simply degraded by any attempt to subordinate it to petty social distinctions. We may rather be proud to consider our work above such, conferring honour, not honoured through the individual. The queen of nurses, Florence Nightingale, has said: "Nursing is an art . . . requiring as hard a preparation, as exclusive a devotion as any painter's or sculptor's work. For what is the having to deal with dead canvas or cold marble compared to the living body, the temple of God's Spirit? It is one of the fine arts, I had almost said the finest of the fine arts." An artist is of no class. In conclusion, I also thank "A. G." for her reminder to "A Doctor's Daughter" that the Great Physician, Whose servants we are, was Himself of humble birth and station, and I would commend to all nurses, especially to all young nurses, the following dedicatory prayer:—

"Most blessed Lord, Who went among the sick
With healing touch, and thus upon Thee took
Our sad infirmities: Grant we may see
Our high prerogative in following Thee
In this our nursing ministry."

*** This controversy is now closed. We thank our numerous correspondents for their letters, several of which we are now unable to print.—ED. *T. H.*

### NEW REGULATIONS FOR NURSING THE PLAGUE.

"A MATRON" writes: Will you allow me as a nurse who has spent a good many years abroad to say that I think it is hardly fair to so severely criticise the rules made for the plague nurses in Calcutta without knowing the special circumstances under which they were made. If I were living in a town in which rioting was taking place I should feel grave anxiety about my nurses if they went out at night unprotected, and I should certainly consider myself obliged to ascertain their destination and the sufficiency of their escort. But even apart from rioting, the whole social atmosphere is so different in India, especially in large towns like Calcutta, that I doubt very much if the liberty rightly accorded to women working at home would be understood there. I believe, for example, that in Calcutta anyone who likes to call upon anyone else is at liberty to do so, and in a country where a household of young women (apart from missionary settlements) is a complete novelty, I can quite understand that the authorities may have feared that some most undesirable people might take advantage of the nurses' ignorance of Indian society, and some most undesirable visitors admitted. It was certainly hard upon the lady doctor to place upon her the onus of refusing to admit some visitors, and of defending her nurses from such undesirable acquaintances, but as she was requested by the authorities to act as superintendent of the nurses the position probably brought her more into touch with the authorities and the residents who knew the place and the people, and could advise her in such matters. To criticise the authorities and to blame them for insulting the nurses is to put a weapon into the hands of those who wish to insinuate that such rules were called for by the conduct of the nurses themselves. I cannot think for a moment that anyone wished to insult women who had come out at the call of duty, and at considerable risk to themselves. The harm done was by the indiscretion of one or more amongst themselves or their friends by rushing into print instead of using proper means to have any rules which pressed hardly upon them altered. They were only intended to be in force for a short time, and as soon as the rioting ceased were withdrawn. The correspondence gave annoyance to many of the nurses, who were thus forced into publicity. I may add that I think the greatest care ought to be exercised in the choice of nurses sent abroad to any position, and that there ought to be an experienced committee (not entirely composed of men) to choose them, and that the nurse's personal as well as professional qualifications ought to be strictly investigated, and no one chosen unless the matron under whom she is working can personally recommend her for such a post. Testimonials, as all matrons know, are of little use. I know of one nurse who was chosen for plague duty, and the matron under whom she was working told me she was never asked anything about her; if she had been consulted she would certainly have not recommended her for such a post.

*** We do not think that the nurses objected to the discipline; in fact, both in their training and their daily work all hospital nurses are accustomed to a discipline which is in many cases very strict. But the officer by whom control is exercised is a "sister" or a superintendent, who has gone through the same mill, and understands a nurse's wants and feelings. The mistake was to make the lady doctor undertake the work of the superintendent. It must not be forgotten that the age for entering as a medical student may be as young as 17, so that by about 23 a young woman may be a qualified medical practitioner, and that such a lady, however clever in her profession, may know absolutely nothing about the requirements of nurses, and have had no experience whatever of governing a community.—ED. *T. H.*

### Notes and Queries.

**The contents of the Editor's Letter-box have now reached such unwieldy proportions that it has become necessary to establish a hard and fast rule regarding Answers to Correspondents. In future, all questions requiring replies will continue to be answered in this column without any fee. If an answer is required by letter, a fee of half-a-crown must be enclosed with the note containing the enquiry. We are always pleased to help our numerous correspondents to the fullest extent, and we can trust them to sympathise in the overwhelming amount of writing which makes the new rules a necessity.**

**Every communication must be accompanied by the writer's name and address, otherwise it will receive no attention.**

*How to Become a Nurse?*

(243) Is there a later edition of Honnor Morten's "How to Become a Nurse"? I want later particulars of the Great Northern Hospital.—*F. E. C.*

"Burdett's Official Nursing Directory" is the latest published, and contains full particulars. Price 5s., from the Scientific Press, London.

*Patient Wintering Abroad.*

(244) I am desirous of finding a patient, who requires a nurse, to winter abroad. Could you please inform me as to the best means of obtaining such a case? I am fully trained and certificated, also had experience in private nursing.—*S. E. F.*

The best way to obtain such a patient is by advertising in medical journals and daily newspapers.

*Sanitary Examination.*

(245) What examination has to be passed for a sanitary inspectorship? I am anxious to hold such a post, and am a three-year certificate nurse.—*Biddy.*

Write to the Secretary, the Sanitary Institute, Margaret Street, W., for particulars.

*The Benevolent Fund.*

(246) I have been in bad health for the last three months, and quite unable to work; the doctor attending me has given me permission to try an easy case. Can you tell me if the Hospital Benevolent Fund would assist me—by a small pecuniary advance—to procure a few necessaries for my work? I am a subscriber to the fund mentioned.—*A Subscriber.*

See rules to correspondents. THE HOSPITAL Convalescent Fund, to which you are possibly referring, is for the purpose of assisting nurses who require change and rest after illness. It cannot make monetary advances.

*Certificate.*

(247) Holding a certificate of training from a recognized training school for 17 months, am I bound to go to same hospital again should I wish to make it three years, viz., in order to be recognized as qualified for promotion under the new Poor Law requirements?—*Mary H.*

There are a few hospitals which will recognize previous training, and, at the end of the period needed to complete the three years, give a three-year certificate. The Victoria Park Hospital for Diseases of the Chest is one. The matron, however, requires that the short-time probationer shall serve two years in that hospital before giving the three-year certificate. Before entering upon any engagement have the terms clearly stated in writing.

*Claim for Nursing Fees.*

(248) Some time ago I refused to attend a confinement, and gave the woman the address of another nurse. In spite of this I was called in after the birth, and could not refuse as the patient was unattended, and I nursed the case for a week. The patient's sister was present, and I being informed that she was helpless with rheumatism in the knee I helped her to bed, and in the dusk rubbed it with embrocation. In the morning I discovered she was suffering from erysipelas, and as she had occupied the same bed as the patient, lent her my chair bed, which has been returned to me in a dirty condition. Can I recover nursing fees for the week, and the value of the damage to my property? I may say that I got blood poisoning from rubbing the knee, which laid me aside for two months.—*Bolton.*

The case as stated is certainly one for which you can claim fees. You should consult a respectable solicitor upon the matter.

*Disinfecting Bed pan.*

(249) Where, in London or Brighton, could I get a rubber bed-pan thoroughly disinfected? It has been used a few times only, but by a cancer patient.—*Nurse Mary.*

The great disadvantage of soft rubber-bedpans is the difficulty of disinfecting them. If thoroughly vulcanised they will bear considerable heat, and may be washed with boiling water; but probably the safest plan is to have them well scrubbed with perchloride of mercury solution (1 in 1,000). We should be glad to have the opinion of hospital sisters on this subject.

### ANSWERS REQUESTED.

*Spinal Curvature.*

(250) We refer "J. H." to our rules anent name and address.

*Heat Poultices and Packs.*

(251) Will some of your matron readers kindly tell me how to apply dry heat, the best way of making an ice pack and ice poultice, and how to make a bran poultice; also a vinegar and bran poultice?—*Jane E. S.*

*To Keep Mattresses.*

(231) The Matron of the Royal Berks Hospital, Reading, sends the following useful information in reply to the query of a correspondent: I personally see the mattresses and condemn them. I then have them fumigated, after which the hair is removed from the ticking cover, and teased out on a "carding" machine, which gets rid of the dust, &c. The covers are, if good enough to repair, washed and mended, or new ticking covers prepared, and an upholstress comes in by the day and makes up the new mattresses with the old hair, for which she requires a mattress needle, strong linen thread, and some chamois leather. I would be pleased to give further information (how I purchase hair, &c.) to anyone who wishes to write personally to me on this subject.

